# Chromosome-level genome assembly of *Yamadazyma mexicana* NCYC 1483

**DOI:** 10.1128/mra.00680-26

**Published:** 2026-07-27

**Authors:** Adrian S. Turner, David J. Baker, Steve A. James, Maria Monzon, Carmen Nueno-Palop

**Affiliations:** 1 National Collection of Yeast Cultures (NCYC), Quadram Institute Bioscience 7308, Norwich, United Kingdom; 2 Food, Microbiome and Health Programme, Quadram Institute Bioscience 7308, Norwich, United Kingdom; 3 Centre for Microbial Interactions, Quadram Institute Bioscience 7308, Norwich, United Kingdom; Rochester Institute of Technology, Rochester, New York, USA

**Keywords:** *Yamadazyma*, *mexicana*, NCYC 1483, chromosome level, WGS, assembly

## Abstract

*Yamadazyma mexicana* is an ascomycetous yeast with industrial utility. Here, we report the near-chromosome resolution genome sequence of *Yamadazyma mexicana* NCYC 1483, the species type strain.

## ANNOUNCEMENT

*Yamadazyma mexicana*, formerly *Pichia mexicana*, is an ascomycetous yeast that has been isolated from a variety of sources, including cacti, soil, insect frass, and forest trees (1). It has recently been identified as a rare opportunistic human pathogen and causative agent of keratitis (2). It also has agricultural utility as a biocontrol agent to suppress post-harvest anthracnose in field-grown avocados, a fungal disease of global economic importance (3). Here, we used long-read sequencing to assemble to near-chromosome resolution the genome sequence of *Y. mexicana* NCYC 1483, the species type strain. This species was originally isolated multiple times during several cactus yeast surveys, conducted between 1974 and 1976, from necrotic tissue (“rot pockets”) of organ-pipe cacti (*Stenocereus therberi*) collected in Arizona and in Sonora and Baja California, Mexico. The species is heterothallic (anamorph *Candida terebra*), and upon mixing of appropriate mating types, zygotes are developed with hat-shaped ascospores (4). The strain here discussed was deposited into the NCYC collection in 1983 by Herman J. Phaff. The strain has equivalent designations in other culture collections as ATCC 42175, CBS 7066, CCRC 22207, JCM 1835, NRRL Y-11818, NRRL Y-11991, UCD 76-308A, and UCD 76-308.1. An Illumina WGS assembly of NRRL Y-11818 has previously been deposited in GenBank (GCA_030569415.1), consisting of 55 contigs (genome size = 11.8 Mb).

Genomic DNA was isolated using a Monarch Spin gDNA extraction kit (New England Biolabs) from cells harvested from a 2-day liquid YM culture (10 g/L glucose, 3 g/L malt extract, 5 g/L peptone, and 3 g/L yeast extract) grown at 25°C. A Nanopore library was prepared from this gDNA prep, without fragmentation or size selection, using the SQK-LSK114 Ligation Kit (Oxford Nanopore Technologies [ONT]), and sequenced on a PromethION P2 solo instrument (ONT) with a FLO-PRO114M flow cell. Default parameters were used for all software unless otherwise specified. Base calling was performed using the super-accurate (v5.0.0, 400 bp) basecall model. This produced 9,697,024 reads with a mean length of 2,041 bases (~1,800× coverage). Read quality was verified with Nanostat (v1.1.2) (5). *De novo* assembly of Nanopore reads was performed using Canu (Galaxy v2.2 [6]) in “nanopore raw” mode with the minimum read length set to 5,000 bp. The final assembly consists of eight contigs, including seven chromosome-sized contigs, all with a 17-bp putative telomeric repeat sequence (5′-ACACCAGACACACCCTT-3′)_n_ identified using Tandem Repeats Finder (v4.09) (7) at each end (Table 1). The total length is 11,923,321 bp, with an N_50_ of 2,198,833 bp and a G + C content of 41.83%. Genome completeness was estimated at 99.2% using BUSCO (v5.5.0) (8) with the saccharomycete_odb10 data set. Augustus (v3.2.3) (9) predicted 5,936 protein-coding genes using the *Saccharomyces cerevisiae* training data set. A comparison of our ONT-derived assembly with the previously deposited NRRL Y-11818 assembly (above) using FastANI (Galaxy v1.3) (10) showed the two assemblies have an average nucleotide identity of 99.9953%.

**TABLE 1:** Genome characteristics of *Yamadazyma mexicana* NCYC 1483

Chromosome #	Canu scaffold #	Size (bp)	Left end^*a*^	Right end^*a*^
1	tig00000001	2,761,830	Telomere (17)	Telomere (17)
2	tig00000002	2,201,598	Telomere (13)	Telomere (14)
3	tig00000003	2,198,833	Telomere (18)	Telomere (14)
4	tig00000004	1,738,877	Telomere (19)	Telomere (19)
5	tig00000005	1,467,447	Telomere (17)	Telomere (25)
6	tig00000006	896,018	Telomere (16)	Telomere (19)
7	tig00000007	606,289	Telomere (7)	Telomere (20)
Mitochondrial	tig00000008	52,429	N/A (circular)^*b*^	N/A (circular)

^*a*^ The number of telomeric repeats is shown in parentheses.

^*b*^ N/A, not applicable.

## Data Availability

This whole-genome shotgun project has been deposited in DDJB/ENA/GenBank (BioProject number PRJEB108795, assembly accession number GCA_984568805/CFHEQV010000000). The raw reads are available in the NCBI Sequence Read Archive under accession number ERR16913441. The predicted Augustus protein coding data set has been deposited in Zenodo (https://doi.org/10.5281/zenodo.20312473).
